# German Chamomile (*Matricaria chamomilla*) Induces Cytochrome P450 Expression Through Increased BMAL1 Protein Expression in Liver Nuclei

**DOI:** 10.1007/s10528-025-11260-7

**Published:** 2025-10-15

**Authors:** Moka Ikeda, Yuya Tsurudome, Mai Enrin, Yukiyo Wada, Michiko Horiguchi, Kentaro Ushijima

**Affiliations:** 1 Division of Pharmaceutics, Faculty of Pharmaceutical Sciences, Sanyo-Onoda City University, Yamaguchi, 756-0884 Japan; 2 Department of Regenerative and Therapeutic Medicine, Graduate School of Medicine, Dentistry and Pharmaceutical Sciences, Okayama University, Okayama, 700-8530, Japan

**Keywords:** Circadian clock, German chamomile, Xenobiotic transporter, Metabolic enzyme, Clock gene

## Abstract

German chamomile (*Matricaria chamomilla*) is a medicinal herb that promotes improved digestion and reduces insomnia. Although it is widely used worldwide, the mechanism of induction of drug-metabolizing enzymes is unknown. We found that German chamomile extracts induced cytochrome P450 expression at the transcriptional stage. *Cyp3a11* expression is decreased at night in wild-type mice, but German chamomile extract induced nocturnal *Cyp3a11* and *Cyp1a2* expression. German chamomile extract increased the nuclear protein expression of the clock gene BMAL1, which drives and abolishes the rhythm of *Cyp3a11* expression. By contrast, German chamomile extract did not significantly alter clock gene expression in the suprachiasmatic nucleus (SCN). Similarly, it did not affect the mRNA expression of the clock genes in the kidneys. Because it did not induce the mRNA expression of ATP-binding cassette (ABC) transporters (*Abcb1a*, *Abcc2*, *Abcc4*, and *Abcg2*) in the kidney, German chamomile extract had no effect on the transcription of pharmacokinetics-related molecules other than CYPs. German chamomile extract promoted liver-selective nuclear transfer rhythm changes in clock genes and induced the expression of CYPs. This study may help to explain the mechanism of drug interactions associated with chronic German chamomile extract consumption.

**Supplementary Information:**

The online version contains supplementary material available at 10.1007/s10528-025-11260-7.

## Introduction

Medicinal herbs are widely used folk remedies worldwide that are used to promote health, prevent disease, and ameliorate illness (Pan et al. 2014). Many medicinal herbs are expected to have antioxidant, sleep-improving, and sedative effects, and the scientific evidence and safety of these herbs have been summarized in a publication by the American Herbal Products Association (Academies 2005; Andalib et al. 2011; Ravipati et al. 2012; Feizi et al. 2019). In addition, the consumption of medicinal herbs can affect the pharmacological actions and pharmacokinetics of drugs (Rombolà et al. 2020; Surana et al. 2021). Habitual consumption of medicinal herbs has been reported to affect the function of normal organs and to cause adverse events such as liver and kidney damage (Amadi and Orisakwe 2018; Xu et al. 2020). Therefore, it is necessary to analyze the effects of medicinal herbs on the physiological functions of normal tissues.

Medicinal herbal extracts can alter the gene expression levels of enzymes involved in detoxification and their enzymatic activities. St. John’s wort decreases the blood levels of its substrate drugs by increasing the gene expression of CYP3A4 and CYP2C19 (Piscitelli et al. 2000; Wang et al. 2004; Frye et al. 2004). Valerian extract inhibits the enzymatic activity of UGT1A1 (Alkharfy and Frye 2007). These changes in the function of hydroxyl detoxification enzymes affect the profile of substances in vivo in the liver. Although the specific mechanism of herb-induced hepatitis is unknown, changes in the biomolecular profile may be a cause of hepatitis.

Clock genes have long been known to be associated with factors that alter the expression of CYPs. Cyp3a11 in the liver (corresponding to CYP3A4 in humans) is regulated by BMAL1 and forms a 24 h expression rhythm (Lin et al. 2019). The expression rhythm of *Cyp3a11* is abolished in *Bmal1* knockout mice (Lin et al. 2019). CYP1A2, which plays a central role in the metabolism of dioxin, has a significant circadian rhythm with a peak in the second half of the stated period (Lu et al. 2013). *Cyp1a2* expression is regulated by the clock gene *NPAS2*, which forms a transcriptional rhythm through the time-dependent binding of the NPAS2–BMAL1 complex to the response sequence (He et al. 2022). This finding indicates that CYPs with the greatest influence on xenobiotics are under the control of BMAL1.

Several small molecules in medicinal herbs have been reported to affect the expression of clock genes. Silybin A in *Silybum marianum* prolongs the function of clock genes by binding to CRY1 and CLOCK (Bian et al. 2022). Piperine in black pepper reverses the decreased expression of clock genes caused by fat deposition diseases (Zhang et al. 2022). These studies indicate that medicinal herbs may increase the expression of clock genes and may also indirectly upregulate the expression of clock gene regulatory molecules. However, no medicinal herbs have been found to directly affect BMAL1 expression. Because of this, no studies have investigated the effects of medicinal herbs–clock gene expression changes–CYP expression rhythm changes.

German chamomile is a medicinal herb used to improve gastrointestinal function and sleep disorders (Srivastava et al. 2010). *Matricaria recutita*, a member of the Asteraceae family, is an annual herb whose white flowers are the medicinal part (Zadeh et al. 2014). Extracts, teas, and essential oils extracted from the flowers are distributed worldwide and are used as common folk remedies (Wilkinson et al. 1999; Anderson et al. 2000; Mazokopakis et al. 2005). Moreover, German chamomile extract is a medicinal herb that may induce some CYPs, which may lead to drug interactions. However, the mechanism by which it induces CYP expression is unknown. We investigated whether drinking German chamomile alters the expression of CYPs, targeting the rhythm of CYP expression. We then observed the expression rhythms of clock genes in response to German chamomile consumption, with the aim of demonstrating the relationship among “German chamomile–changes in the expression of clock genes–expression rhythms of CYPs” and verifying this relationship.

## Methods

### Animals

Six-week-old male C57BL/6 J Ham Slc- + */* + mice were purchased from Japan SLC Inc. (Shizuoka, Japan). Mice were housed in a light-controlled room at a temperature of 24 ± 1 ℃ and 60% ± 10% humidity, with food and water available ad libitum. In the light/dark cycle, the zeitgeber times (ZTs) of ZT0 and ZT12 were defined as the times for lights on and off, respectively. All mice were held for 2 weeks to acclimatize before the treatment. During the dark period, a dim red light was used to aid in animal treatment. All the experiments were conducted in accordance with a protocol approved by the internal committee for animal experimentation in Sanyo-Onoda City University (ethical approval protocol IC: #A-2023–44-A).

### Preparation of Extracts from German Chamomile

Dried flower heads of German chamomile (*Matricaria chamomilla)* were obtained from Herb Meister Center (Tokyo, Japan). German chamomile was harvested in March 2021 in Japan. The extract was prepared immediately after purchase (September 2021). Extracts of German chamomile were prepared by suspending the 5.0 g of finely cut dried flower heads in 150 mL of 99.5% ethanol and periodic sonication for 15 min at room temperature. The mixture was filtered through absorbent cotton. The filtrate evaporated under reduced pressure (60–90 hPa) at 45 ℃ for 10 min using rotary evaporator. The residue was redissolved in 10 mL of 99.5% ethanol and filtered and stored at − 30 ℃ until further use. To assess the stability of the extract, HPLC analysis was conducted using the following three preparations: (i) freshly prepared extract, (ii) extract after concentration with rotary evaporation at 45 ℃, and (iii) extract after concentration followed by storage at − 30 ℃ for one week. We checked that most of the major constituents were retained in the GC extract even after storage at – 30 ℃ (Supplementary Fig. 1).

### Treatment of Mice with German Chamomile Extract via Drinking Water

**Fig. 1:**
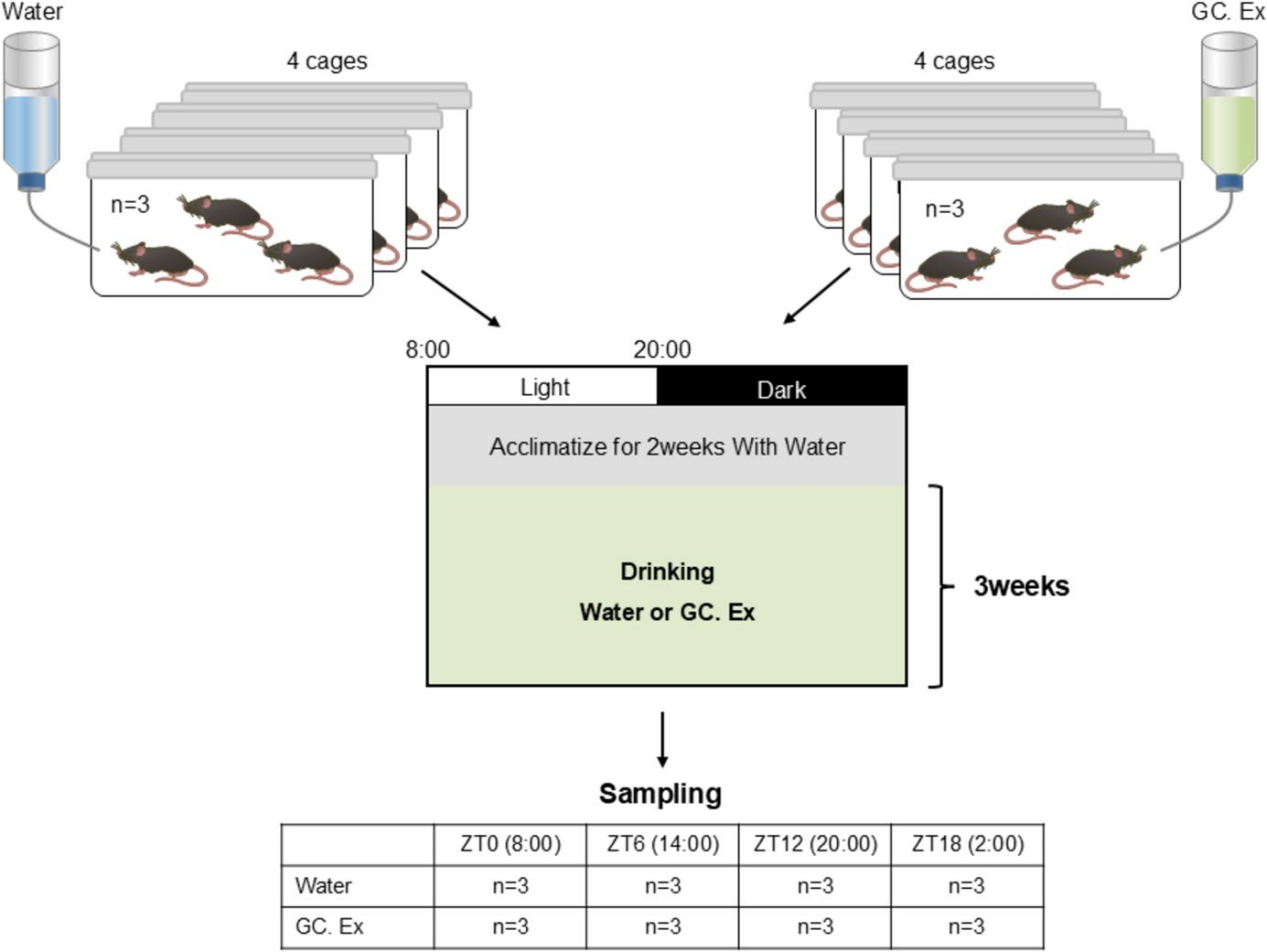
Schematic illustration of the animal experimental design. Mice were randomly assigned to the vehicle or German chamomile extract (GC Ex.) groups. Each group included 12 mice, and three mice were used at each time point. GC Ex. was administered in drinking water for 3 weeks, and tissue collection was performed at designated time points

After acclimation, 24 mice were randomly assigned to two groups (vehicle group (n = 12) and German chamomile extract (GC Ex.) group (n = 12)). Three mice were used at each time point; thus, 12 mice were needed per group. Drinking water containing 2% (v/v) ethanol and 2% (v/v) ethanol dissolved with German chamomile extract at a final concentration of 0.05% (w/v) received oral administration into vehicle group and GC Ex. group, respectively, during the period of 3 weeks. No differences in water consumption and food intake changes were observed between the two groups at the 3-week mark (Supplementary Fig. 2). To facilitate understanding of the experimental design, a schematic illustration was created and is provided in Fig. 1.

### Tissue Isolation

Each mouse was placed in a chamber filled with isoflurane, and the whole brain and blood were collected under hyperanaesthesia. At four time points with an interval of 6 h starting at ZT0, the brain, liver, and kidney of each mouse were harvested. Isolated brains were sectioned into slices using a brain matrix (BS-Z 2000 C; Muromachi Kikai Co. Ltd., Tokyo, Japan). The suprachiasmatic nucleus (SCN) was cut from the slice sections and used for RNA extraction experiments.

### RNA Isolation and Quantitative Real-Time Polymerase Chain Reaction (qRT-PCR)

Total RNA was extracted from the mouse tissue using the PureLink® RNA Mini Kit (for SCN; Thermo Fisher Scientific, Waltham, MA, USA) and RNAzol RT reagent (for liver and kidney; Molecular Research Center, Inc., Cincinnati, OH, USA) according to the manufacturers’ instructions. Reverse transcription was performed with the PrimeScript™ RT reagent Kit (Takara Bio, Otsu, Japan). The cDNA equivalent of 5 ng of RNA was amplified by PCR using a StepOnePlus™ Real-Time PCR System (Life Technologies, Carlsbad, CA, USA) with TB Green® Premix Ex Taq™ Ⅱ (Takara Bio). The reaction was first incubated at 95 ℃ for 30 s, followed by 40 cycles at 95 ℃ for 5 s and 60 ℃ for 30 s. All experiments that used kits were performed according to the manufacturer’s instructions. The comparative Ct method was applied to calculate the relative mRNA expression levels. The data were normalized to the 18S ribosomal RNA gene (*Rn18s*) used as the internal control. The nucleotide sequences for the specific gene primers used in this study are listed in Table 1.

**Table 1:** Primer sets for qPCR analysis of gene expression

Gene name	Accession ID	Primer	Sequence
*Arntl*	NM_007489	F	ACGACATAGGACACCTCGCAGA
		R	CGGGTTCATGAAACTGAACCATC
*Clock*	NM_007715	F	AACCGTAGCAGGTTTATGGGAATG
		R	TTGGTGTCCACACAATAGGCAAG
*Cry1*	NM_007771	F	GGATCCACCATTTAGCCAGACAC
		R	CATTTATGCTCCAATCTGCATCAAG
*Dbp*	NM_016974	F	AAGCATTCCAGGCCATGAGAC
		R	TTCTTGTACCTCCGGCTCCAG
*Per1*	NM_011065	F	GTCTGGTTCAGGATCCCACGA
		R	TGCTGCCAAAGTACTTGCTTGTATG
*Per2*	NM_011066	F	ATCAGCCATGTTGCCGTGTC
		R	CGTGCTCAGTGGCTGCTTTC
*Cyp1a2*	NM_009993.3	F	CGTCAGCAAGCTTCAGAAGG
		R	CGATGTTCAGCATCTCCTCG
*Cyp3a11*	NM_007818.3	F	GCCATTTTTAGGCACTGTGCTGA
		R	TGTGACAGCAAGGAGAGGCGT
*Rn18s*	NR_003278.3	F	CGGCTACCACATCCAAGGAA
		R	GCTGGAATTACCGCGGCT

### Nuclear Protein Extraction

Hepatic nuclear proteins were extracted from the mouse livers using the FractionPREP™ cell fractionation kit (K270; Biovision, Mountain View, CA, USA) according to the manufacturer’s instructions (Canesin et al. 2021). Briefly, each tissue sample was minced using a scalpel and washed with ice-cold PBS twice (pH 7.4; Thermo Fisher Scientific). Samples were homogenized using a Potter–Elevhjem tissue grinder in 400 μL of cytosol extraction buffer with dithiothreitol (DTT) and protease inhibitor cocktail. The samples were then incubated on ice for 20 min with gentle tapping three to four times every 5 min after pipetting several times to mix well. The homogenate was centrifuged at 700 × g for 10 min at 4 ℃. Next, 400 μL of membrane extraction buffer with DTT and protease inhibitor cocktail was added to the pellet, the sample was mixed after adding 22 μL of membrane extraction buffer B, and then it was incubated on ice for 1 min. The solution was centrifuged at 1000 × g for 5 min at 4 ℃. Then, 200 µL of ice-cold nuclear extraction buffer Mix with DTT and protease inhibitor cocktail was added to the pellet, it was vortexed for 15 s, and then the sample was kept on ice for 40 min with constant vortexing for 15 s every 10 min. The solution was centrifuged at 20,000 × g for 10 min at 4 ℃. The supernatant was used as the hepatic nuclear fraction.

### Western Blots

Western blots were performed as previously described (Tsurudome et al. 2022). Hepatic nuclear fractions were denatured at 95 ℃ for 3 min with 1% SDS and 5% 2-mercaptoethanol. Denatured samples containing 10 µg of each protein fraction were separated by sodium dodecyl sulfate–polyacrylamide gel electrophoresis (SDS–PAGE) and transferred onto a polyvinylidene difluoride membrane. Separated proteins on a TGX stain-free SDS-gel (Bio-Rad, Hercules, CA, USA) were stained as a control for equal loading of the nuclear fraction proteins. The membranes were blocked with 1% skim milk (#9999; Cell Signaling Technology, Beverly, MA) in Tween 20–TBS at room temperature (20–25 ℃) for 1 h with constant agitation. The membranes were incubated with anti-Cyp3a11 (1:1000; 18227–1-AP; Proteintech, Tokyo, Japan), anti-Cyp1a2 (1:1000; 19936–1-AP; Proteintech), anti-BMAL1 (1:1000; ab15602; Abcam, Cambridge, UK), anti-PER1, and anti-CRY1 primary antibodies diluted with Can Get Signal Solution 1 (Toyobo, Osaka, Japan). Specific antigen–antibody complexes were visualized using HRP-conjugated anti-rabbit IgG (1:10,000; sc-2032; Santa Cruz Biotechnology, Santa Cruz, CA) diluted with Can Get Signal Solution 2 (Toyobo) and ECL Western Blotting Substrates (Bio-Rad). Visualized images were scanned by a BIO-RAD ChemiDoc™ Touch Imaging System (Bio-Rad).

### Statistical Analyses

All data are expressed as the mean ± standard error of the mean (SEM). Statistical analyses were performed using GraphPad Prism software (ver. 8; GraphPad Software, San Diego, CA, USA). Differences among the groups were analyzed by two-way ANOVA, followed by Tukey’s post hoc tests. *P* < 0.05 was considered statistically significant (The results of the analysis are listed in Supplementary Table 1–6). Although no statistical methods were used to predetermine the sample size, the sample sizes used in the present study are similar to those reported in previous studies (Ravipati et al. 2012; Lin et al. 2019; Tsurudome et al. 2022). The experiments were not randomized.

## Results

### Cytochrome P450 mRNA Expression in Mouse Liver is Promoted by German Chamomile Extract

**Fig. 2:**
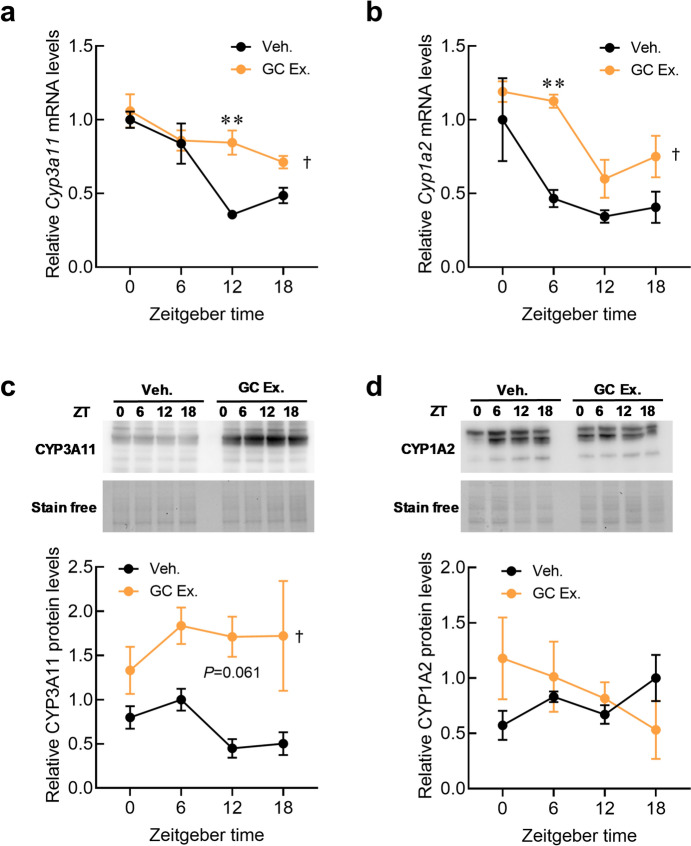
German chamomile extract induces cytochrome P450 mRNA expression in the mouse liver. **a, b** Temporal expression profiles of *Cyp3a11* (**a**) and *Cyp1a2* (**b**) mRNA in in the liver of vehicle and GC Ex. groups. **c, d** Temporal expression profiles of Cyp3a11 (**c**) and Cyp1a2 (**d**) protein in in the liver of vehicle and GC Ex. groups. Stain-Free Gel image indicates the equal loading of proteins from the liver. Values are shown as the mean with S.E.M. (n = 3). The mean value of the vehicle group at the peak time was set as 1. †, *P* < 0.05, significantly different between the two groups; **, *P* < 0.01, significantly different from vehicle group at the corresponding time point (two-way ANOVA with Tukey’s post hoc test)

To determine whether cytochrome P450 expression was increased by German chamomile extract at the transcriptional level, we measured *Cyp3a11* and *Cyp1a2* mRNA expression in the mouse liver. The *Cyp3a11* and *Cyp1a2* mRNA expression levels were significantly higher in the German chamomile extract (GC Ex.) group than in the vehicle group (Fig. 2a, b). The *Cyp3a11* and *Cyp1a2* mRNA expression rhythms in the vehicle group showed a significant diurnal rhythm with higher expression in the light period than in the dark period, similar to the results in previous reports (Lin et al. 2019; Lu et al. 2013). In the GC Ex. group, the *Cyp3a11* mRNA and Cyp3a11 protein expression rhythm was flattened and high even in the dark period (Fig. 2a, c). These results indicate that the expression of CYPs is induced at the transcriptional step by German chamomile extract and that this disrupts the rhythm of CYP expression.

### German Chamomile Extract Upregulates BMAL1 Expression in Mouse Liver Nuclei

**Fig. 3:**
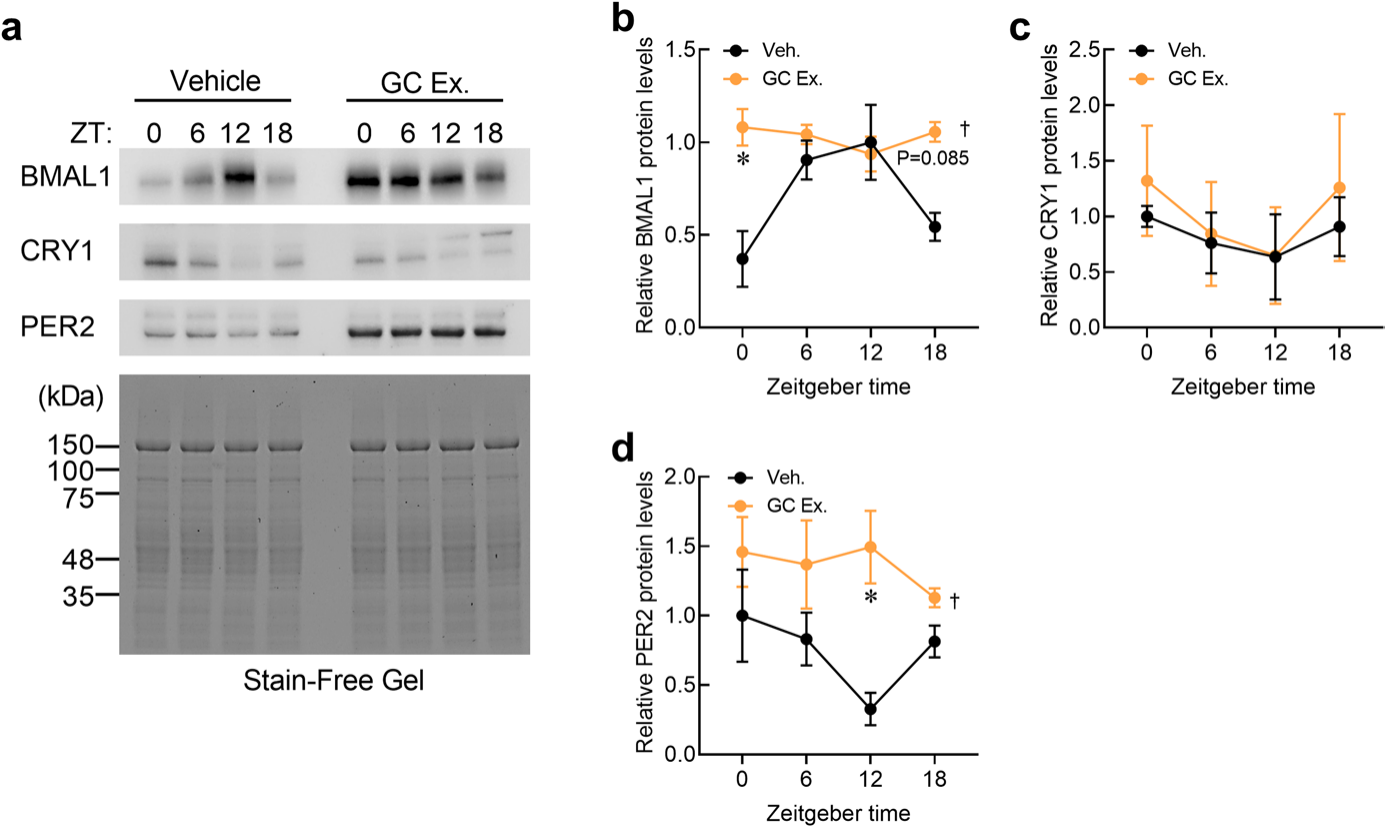
Altered expression of clock gene proteins in the mouse hepatic nuclear region in the German chamomile extract group. **a** The upper panel shows Western blot images, and the lower panel shows a CBB stain image. Stain-Free Gel image indicates the equal loading of proteins from the liver. **b–d** Temporal expression profiles of BMAL1 (**b**), CRY1 (**c**) and PER2 (**d**) protein in the nuclear fraction of vehicle and GC Ex. groups. Values are shown as the mean with S.E.M. (n = 3). The mean value of the vehicle group at the peak time was set as 1. †, *P* < 0.05, significantly different between the two groups; *, *P* < 0.05, significantly different from vehicle group at the corresponding time point (two-way ANOVA with Tukey’s post hoc test).

The rhythm of Cyp3a11 expression is largely mediated by BMAL1 expression (Lin et al. 2019). To evaluate how the German chamomile extract affects the clock mechanism of the liver, we extracted nuclear fraction proteins from the liver and measured nuclear clock gene expression. A distinct circadian rhythm was observed for BMAL1 protein expression in the liver nuclei of the control group (Fig. 3a, b). By contrast, BMAL1 expression in the GC Ex. group did not show the same circadian rhythm as that in the control group. Moreover, nuclear BMAL1 expression was high at all time points. The expression of CRY1 and PER2, which are suppressors of BMAL1, was also measured, although no significant changes were observed because of the large individual differences (Fig. 3c, d).

### Influence of German Chamomile Extract on the Transcription of Clock Genes in Mouse Liver

**Fig. 4:**
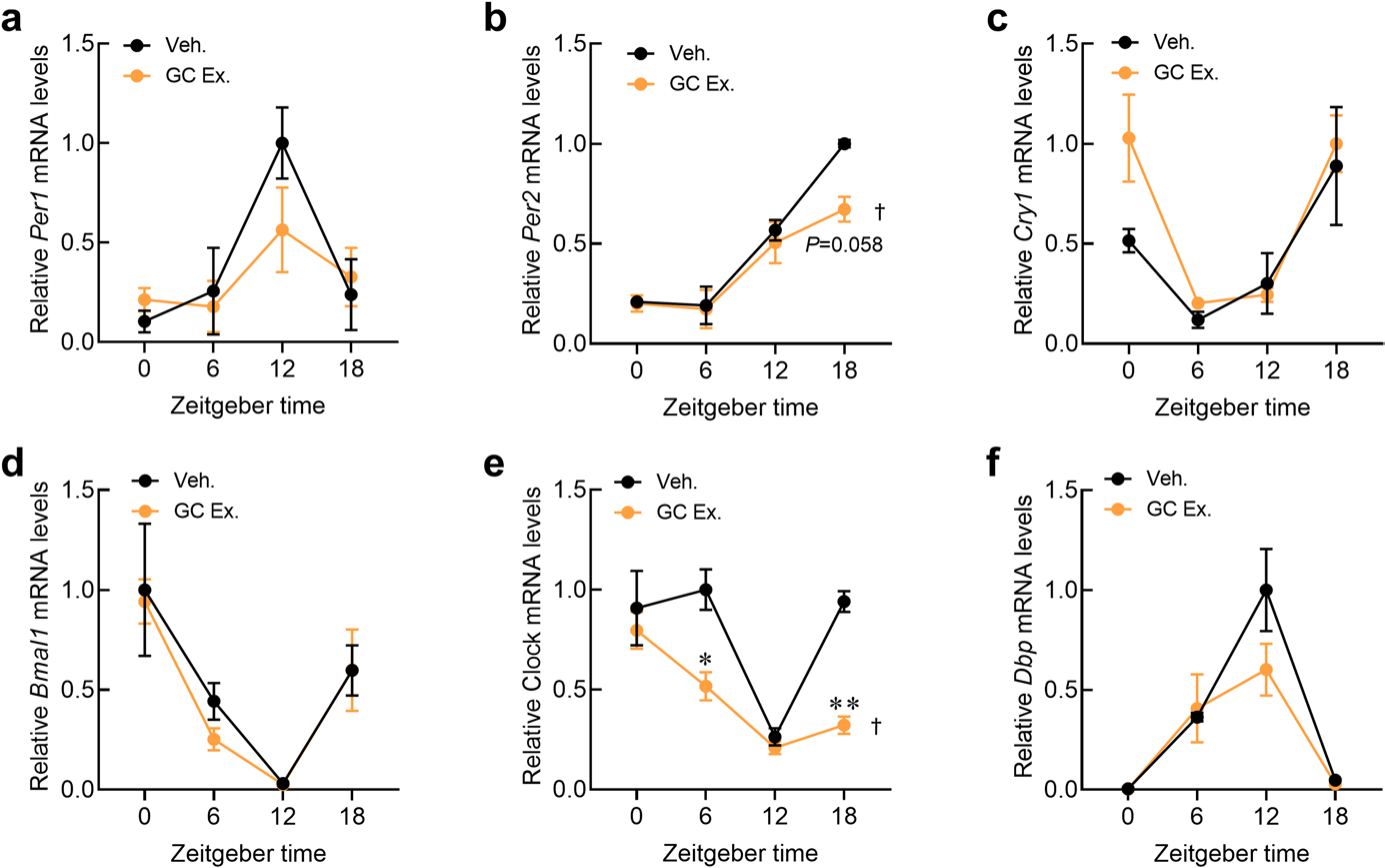
German chamomile extract affect the clock gene expression in the mouse Liver. **a–f** Temporal expression profiles of *Per1* (**a**), *Per2* (**b**), *Cry1* (**c**), *Bmal1* (**d**), *Clock* (**e**), and *Dbp* (**f**) mRNA in the liver of the vehicle and GC Ex. groups. Values are shown as the mean with S.E.M. (n = 3). The mean value of the vehicle group at the peak time was set as 1. †, *P* < 0.05, significantly different between the two groups; *, *P* < 0.05, significantly different from vehicle group at the corresponding time point (two-way ANOVA with Tukey’s post hoc test)

To evaluate how the German chamomile extract affects the clock mechanism of the liver, we measured the clock gene expression rhythm in the liver (Fig. 4). The cycle of *Cry1* mRNA expression was shortened in the GC Ex. group compared with the control group (Fig. 4c, Supplementary Table 3). The results showed that *Clock* mRNA expression in the liver at ZT6 and ZT18 was decreased (Fig. 4e). In addition, the cycle of *Clock* mRNA was prolonged, and its expression was decreased compared with that in the control group. However, the changes in *Bmal1* mRNA expression were not as significant as the increase in the nuclear protein expression of BMAL1. This result suggests that the German chamomile extract did not affect the transcription mechanism of the Clock systems; instead, it affected the post-transcriptional modification process, causing the induction of liver BMAL1 expression.

### German Chamomile Extract has no Effect on Clock Gene Expression in the Mouse SCN

**Fig. 5:**
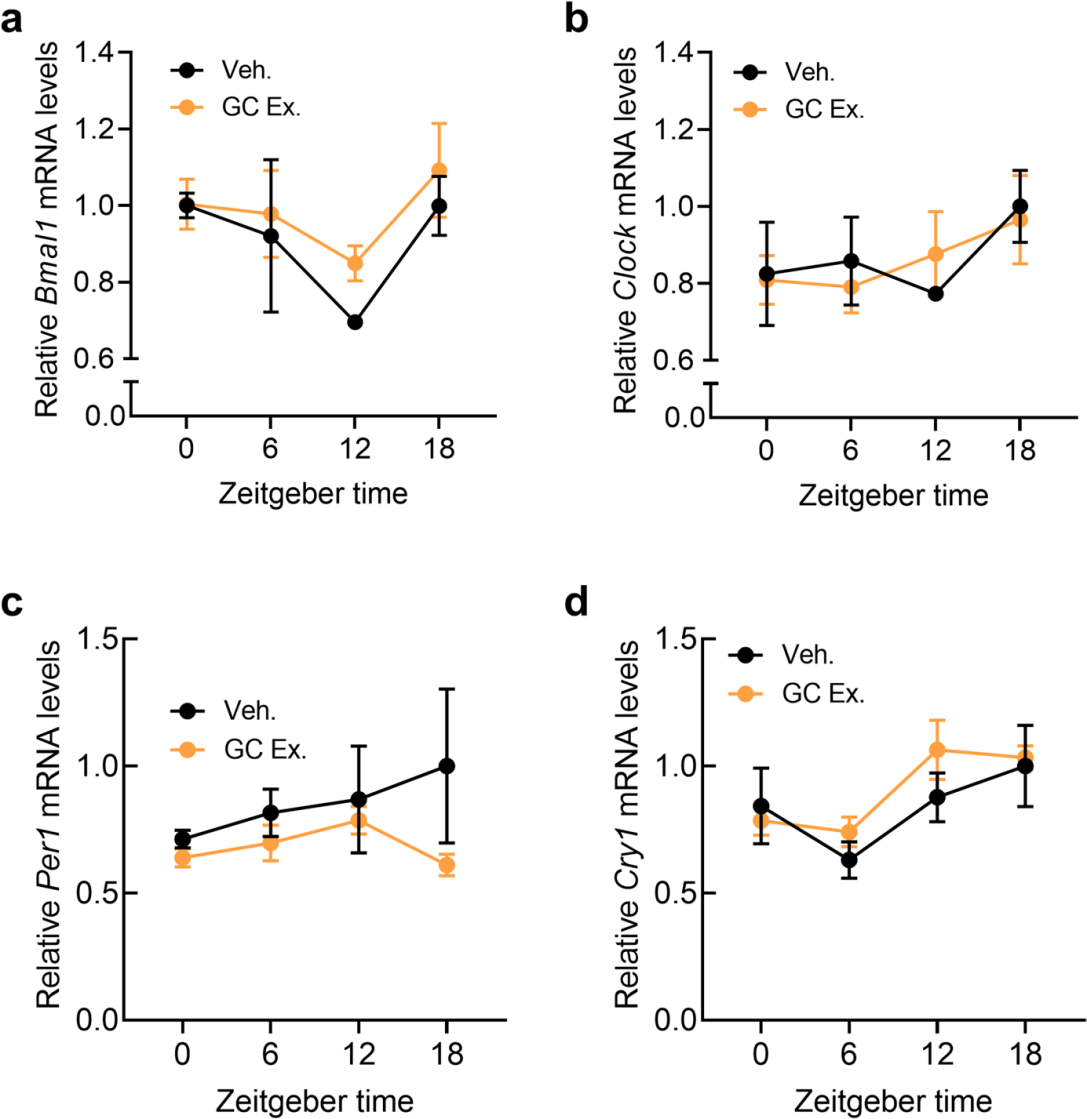
German chamomile extract does not affect the clock gene mechanism in the mouse SCN. **a–d** Temporal expression profiles of *Bmal1* (**a**), *Clock* (**b**), *Per1* (**c**), and *Cry1* (**d**) mRNA in the SCN of the vehicle and GC Ex. groups. Values are shown as the mean with S.E.M. (n = 3). The mean value of the vehicle group at the peak time was set as 1. There was no significant difference between the two groups (two-way ANOVA with Tukey’s post hoc test)

The clock mechanism of the organism is synchronized by transmission from the master clock system in the SCN. To determine whether the German chamomile extract affects the clock system in the SCN, we measured the clock gene expression levels in the SCN. The results showed no significant changes in the rhythm and mRNA expression levels of *Clock*, *Bmal1*, *Per1*, and *Cry1* (Fig. 5a–d). These results indicated that the German chamomile extract affects the peripheral clock mechanism without affecting the central clock mechanism.

***German Chamomile Extract has no Effect on the Kidney Clock Mechanism or the Rhythm of Expression of ATP-Binding Cassette (ABC) Transporters in the Mouse Kidney.***

**Fig. 6:**
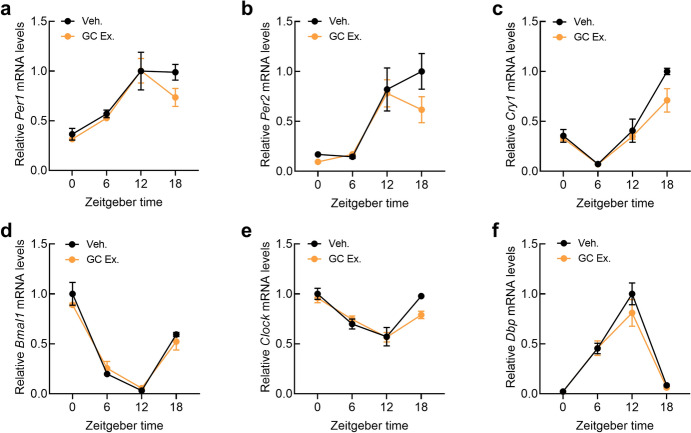
German chamomile extract does not affect the clock gene mechanism in the mouse kidney. **a–f** Temporal expression profiles of *Per1* (**a**), *Per2* (**b**), *Cry1* (**c**), *Bmal1* (**d**), *Clock* (**e**), and *Dbp* (**f**) mRNA in the kidney of the vehicle and GC Ex. groups. Values are shown as the mean with S.E.M. (n = 3). The mean value of the vehicle group at the peak time was set as 1. There was no significant difference between the two groups (two-way ANOVA with Tukey’s post hoc test)

The German chamomile extract causes changes in the peripheral clock mechanism, and therefore it is assumed that other organs would be affected. To analyze whether the German chamomile extract also affected the clock mechanism in other organs, the clock gene expression cycle in the kidney was evaluated. The results showed that there were no significant differences between the two groups, with no changes in the expression cycle of clock genes in the kidneys compared with that in the liver (Fig. 6a–f).

**Fig. 7:**
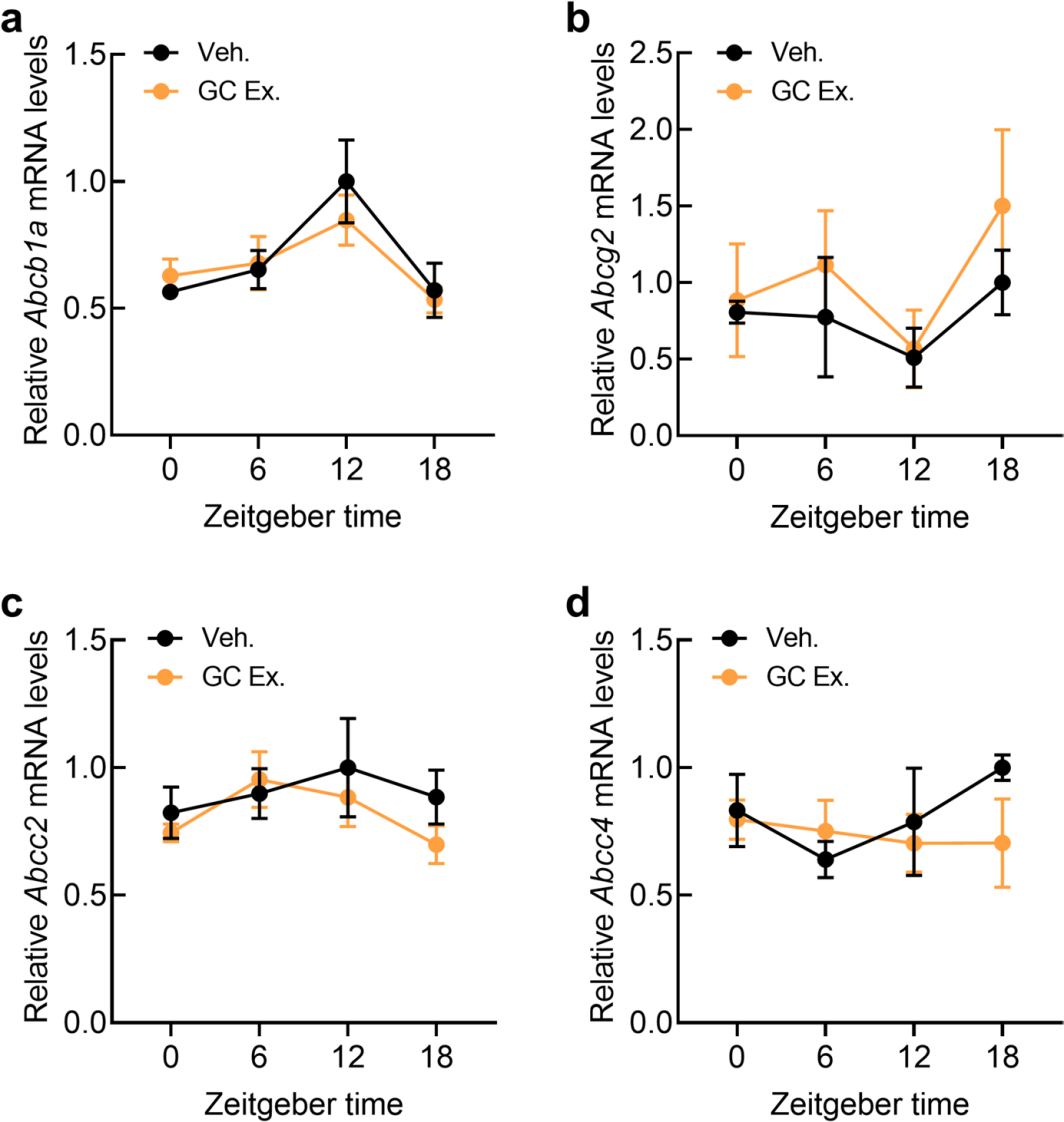
Renal ABC transporters are not induced by German chamomile extract. **a–d** Temporal expression profiles of *Abcb1a* (**a**), *Abcc2* (**b**), *Abcc4* (**c**), and *Abcg2* (**d**) mRNA in the kidney of the vehicle and GC Ex. groups. Values are shown as the mean with S.E.M. (n = 3). The mean value of the vehicle group at the peak time was set as 1. There was no significant difference between the two groups (two-way ANOVA with Tukey’s post hoc test)

Some medicinal herbal extracts induce the expression of ABC transporters. Therefore, we measured the mRNA expression levels of the ABC transporters *Abcb1a*, *Abcc2*, *Abcc4*, and *Abcg2*, which are highly expressed in the kidney. The expression levels of these ABC transporters were not significantly different between the two groups (Fig. 7a–d). Thus, the effects of German chamomile extract on the peripheral clock mechanism are selective for the liver and have little effect on the kidneys.

## Discussion

The mechanism by which German chamomile induces cytochrome P450 expression has not been clarified. The results of this study indicate that German chamomile extract induces the transcription of *Cyp3a11* gene in the mouse liver. The induction of *Cyp3a11* transcription was attributed to increased BMAL1 protein expression in the liver nucleus. German chamomile extract affected the expression cycle of clock genes in the liver. In contrast, it did not significantly alter the clock gene expression cycles in other organs such as the kidney and SCN. These results indicate that the effect of German chamomile extract on clock gene expression cycle is liver-selective, which induces CYP expression.

The induction of drug-metabolizing enzyme expression by medicinal herbs affects pharmacokinetics (Rombolà et al. 2020; Fasinu et al. 2012). St. John’s wort causes the induction of CYP3A4 expression and decreases blood levels of tacrolimus and digoxin (Johne et al. 1999; Obach et al. 2004). The mechanism of increased CYP3A4 expression is mainly due to increased transcriptional activity via nuclear receptors (e.g., PXR and CAR) (Moore et al. 2000; Masi et al. 2009). German chamomile contains high amounts of flavonoids such as apigenin; however, these main components do not activate the PXR pathway (Dong et al. 2010). The increase in *Cyp3a11* expression induced by German chamomile extract observed in this study weakly implicates the widely known PXR pathway.

German chamomile extract and apigenin intake have a significant effect on the lipid metabolism mechanism in the liver. Apigenin accelerates fat uptake, promotes lipid metabolism, and increases NAD + levels (Escande et al. 2013; Jung et al. 2016). Although these physiological effects of apigenin are known, the mechanism is unknown. BMAL1 accelerates lipid uptake in the liver (Gu et al. 2024). Furthermore, NAD + synthase is also regulated by BMAL1 (Nakahata et al. 2009). On the basis of the above information, it is assumed that the previously unknown changes in various physiological activities by apigenin are accompanied by an increase in the nuclear protein BMAL1.

From a pharmacokinetic standpoint, there are no reports indicating clinical concerns related to the increased expression of Cyp3a11 associated with the concurrent use of German chamomile. Conversely, Cyp3a11 has physiological roles beyond drug metabolism, including the hydroxylation of steroid hormones and bile acids. Specifically, testosterone and cortisol undergo hydroxylation at the 6β-position by Cyp3a11 (Ghosh et al. 1995; Meyer et al. 2009). According to pervious report on patients with polycystic ovarian syndrome who were given German chamomile extract in their drinking water, a decrease in plasma testosterone concentration was observed (Afiat et al. 2022). Furthermore, the activity of Cyp3a11 is vital for the regulation of bile acid composition through the hydroxylation of bile acids and chenodeoxycholic acid (Gardès et al. 2013; Chiang and Ferrell 2020). Therefore, the intake of German chamomile extract may promote the metabolism of low molecular weight compounds with a steroid skeleton by increasing the expression of Cyp3a11.

Xenobiotic factor expression induced by German chamomile extract appeared to be limited to the liver rather than the kidney. The liver is an organ that undergoes first-passage effects in the body and is prone to fat-soluble substance deposition. Flavonoid components in German chamomile are more likely to accumulate in the liver (Wilkinson et al. 1999). In contrast, in the kidney, most water-soluble substances are filtered by the glomerulus and transferred to the ureter and may not be taken up by renal tubular cells. Therefore, it is likely that BMAL1 nuclear protein expression in the kidney was not affected, and as a result, no effect on clock gene or ABC transporter expression was observed.

There was no positive control group in this study using extracts previously reported to alter clock gene expression. Although previous studies have shown that certain natural products, such as black pepper constituents and Silybum marianum extracts, can modulate clock gene expression (Bian et al. 2022; Zhang et al. 2022), these effects were observed only at very high doses that are not physiologically relevant to human consumption. Moreover, those compounds appeared to act across multiple organs, whereas our findings indicate that German chamomile extract exerts a more liver-selective influence (Figs. 3, 4, 5, 6 and 7). Therefore, we did not include a positive control in the present study. Future studies incorporating appropriate pharmacological agents or natural compounds with physiologically relevant effects on circadian gene expression will help validate our experimental system.

Another limitation of this study is that we did not perform a comprehensive chemical characterization of the German chamomile extract. While major flavonoids such as apigenin and luteolin are known constituents (Dong et al. 2010; Escande et al. 2013; Ishizaki et al. 2024), we did not conduct a detailed analysis to identify and quantify all active components. This decision was made because the primary aim of the present study was to investigate the overall effect of the extract on clock gene expression, rather than to dissect the contribution of individual compounds. Future studies should include a thorough phytochemical analysis to clarify which specific constituents are responsible for the observed liver-selective effects on circadian gene expression.

The results of this study uncover the mechanism of action of medicinal herbs in terms of changes in clock gene function. We found that German chamomile extract caused an increase in the amount of BMAL1 nuclear protein in the liver and increased the expression of *Cyp3a11*. In addition to CYP expression, BMAL1 is located upstream of genes that exert many nutrient-induced physiological effects, such as fatty acid metabolism, lipid uptake, and glycogen degradation (Gu et al. 2024; Udoh et al. 2018). Elevated nuclear BMAL1 protein levels may be one of the mechanisms by which medicinal herbs alter drug metabolism and physiological functions. Further analysis along the lines of this study will lead to a more detailed understanding of the mechanisms of action of medicinal herbal extracts and promote the appropriate use of medicinal herbs.

## Conclusion

This study demonstrates that German chamomile extract exerts tissue-specific effects on circadian regulation of drug-metabolizing enzymes. The liver-selective disruption of BMAL1-mediated circadian rhythms suggests that German chamomile’s therapeutic benefits are achieved through precise hepatic modulation without globally disrupting the body’s master circadian clock. These findings explain potential drug interactions in patients chronically consuming German chamomile. This contributes to understanding how herbal remedies work at the molecular level.

## Supplementary Information

Below is the link to the electronic supplementary material.

Supplementary file1 (PDF 583 KB)

Supplementary file2 (XLSX 24 KB)

## Data Availability

No datasets were generated or analysed during the current study.
