# Supplementary material for: German Chamomile (Matricaria chamomilla) Induces Cytochrome P450 Expression Through Increased BMAL1 Protein Expression in Liver Nuclei
Source: Biochem Genet. 2025 Oct 15;64(4):4863–79. doi: 10.1007/s10528-025-11260-7 (PMC13388513; doi:10.1007/s10528-025-11260-7)
Supplement: Supplementary file 1 — Supplementary file1 (PDF 583 KB) [file 10528_2025_11260_MOESM1_ESM.pdf]

(a) freshly prepared extract

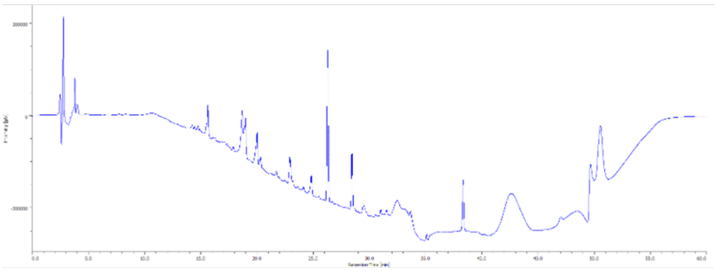

(b) with rotary evaporation at 45℃

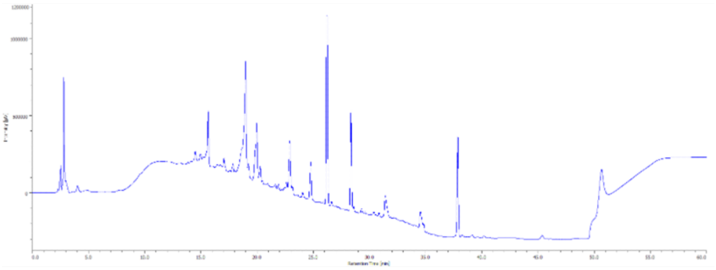

(c) storage at −30℃ for one week

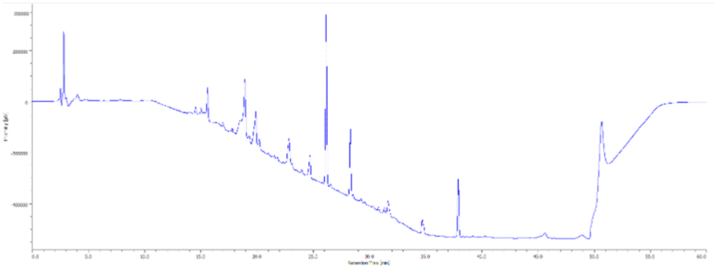

Supplementary Figure 1. Comparison of German chamomile extract profiles under different storage conditions.

HPLC analysis was performed to evaluate the stability of the extract under preparation and storage. Three samples were examined: (a) freshly prepared extract, (b) extract after concentration with rotary evaporation at 45 °C, and (c) extract after concentration followed by storage at -30 °C for one week. The results confirmed that most of the major constituents were retained in the GC extract even after storage at -30 °C.

[HPLC conditions]

Instrument: JASCO LC-NET2/ADC pump with JASCO UV-4075 detector

Column: Mightysil RP-18 GP 250-4.6 (5 µm)

Detection: UV at 210 nm   Flow rate: 1.0 mL/min

Mobile phase: A = acetonitrile, B = MilliQ water with 20 mM ammonium formate

Gradient conditions: 0–5 min, A:B = 10:90; 5–30 min, linear gradient from A:B = 10:90 to 100:0; 30–45 min, A:B = 100:0; 45–50 min, linear gradient from A:B = 100:0 to 10:90; 50-60min, A:B = 10:90

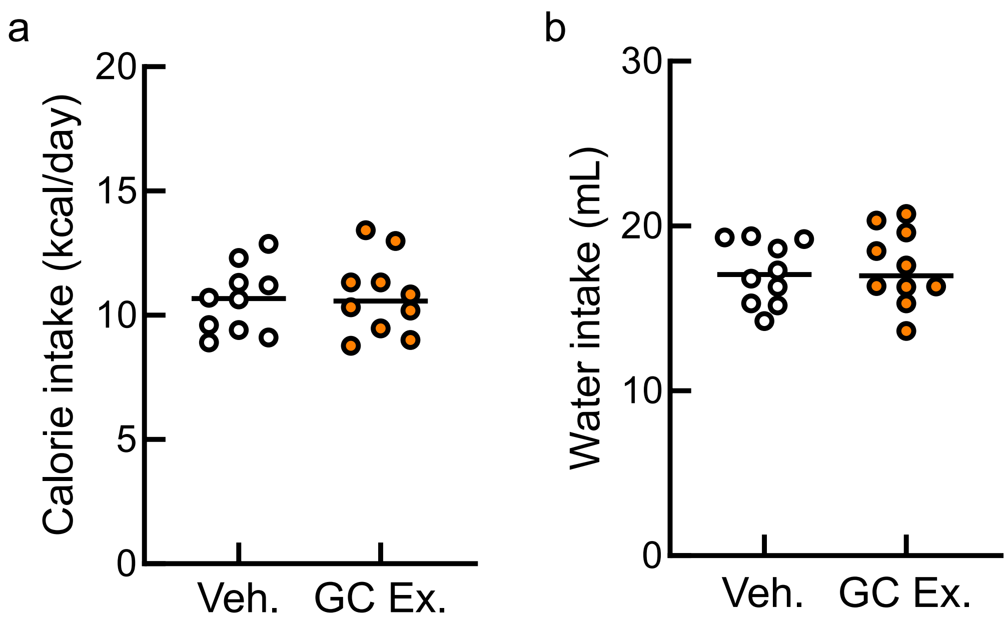

Supplementary Figure 2 Food and water intake were unchanged in the mouse of German chamomile extract group.

(a,b) Twenty-four hour intake of calorie (a) and water (b) were not significantly different ($P > 0.05$) between vehicle and GC Ex. groups that were fed ad libitum after 3weeks.

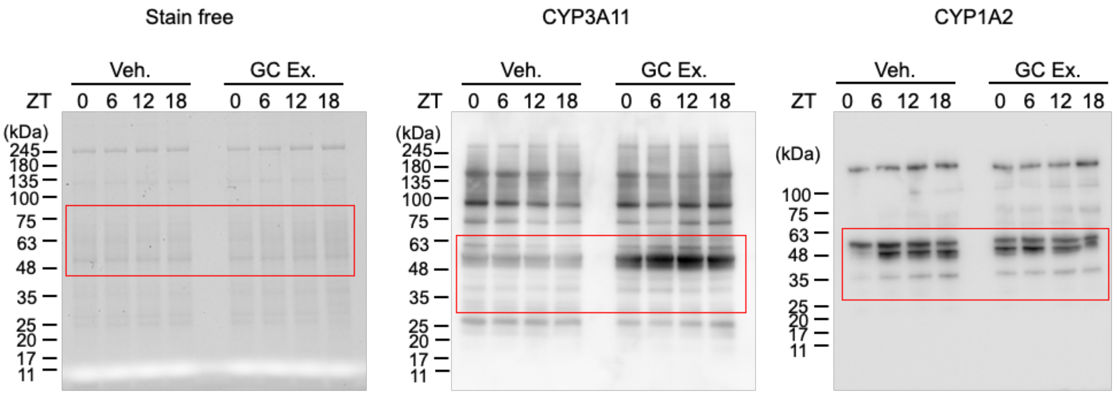

Supplementary Figure 3 Unedited full blots of Figure 2.

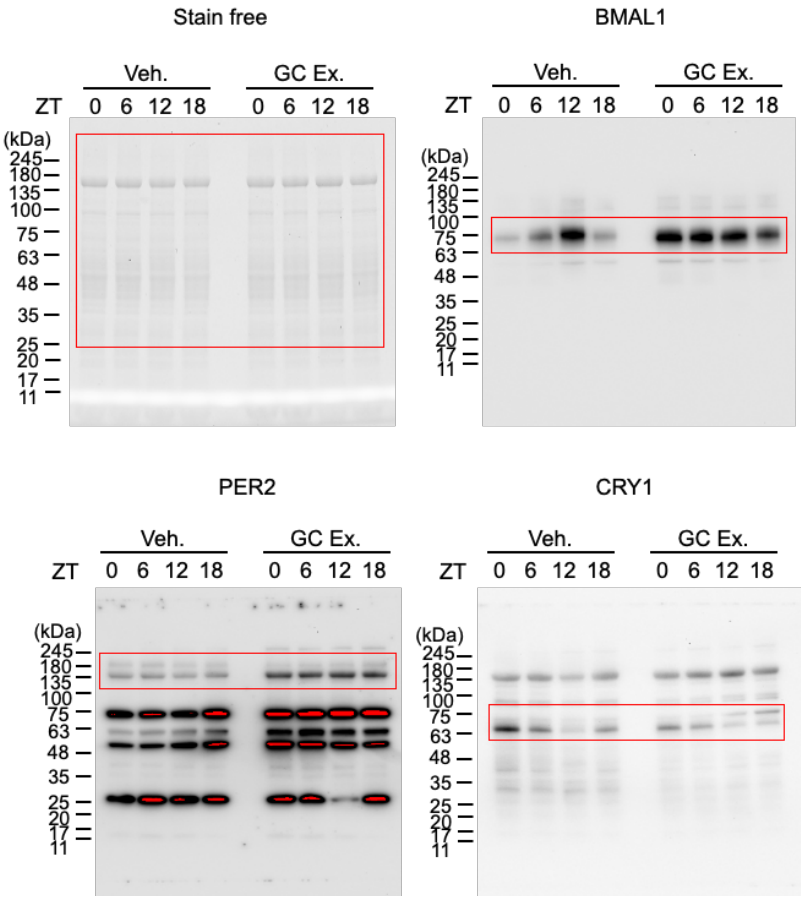

Supplementary Figure 4 Unedited full blots of Figure 3.

The red boxes indicate the regions shown in the corresponding main figure panels. Red-shaded areas denote signal saturation during image acquisition.
